# A novel self-enhanced electrochemiluminescence immunosensor based on hollow Ru-SiO_2_@PEI nanoparticles for NSE analysis

**DOI:** 10.1038/srep22234

**Published:** 2016-02-26

**Authors:** Limin Zhou, Jianshe Huang, Bin Yu, Tianyan You

**Affiliations:** 1 State Key Laboratory of Electroanalytical Chemistry, Changchun Institute of Applied Chemistry, Chinese Academy of Sciences, Changchun 130022, China; 2 University of the Chinese Academy of Sciences, Beijing 100049, China; 3 National Engineering Laboratory for AIDS Vaccine, School of Life Sciences, Jilin University, Changchun 130012, China

## Abstract

Poly(ethylenimine) (PEI) and Ru(bpy)_3_^2+^-doped silica (Ru-SiO_2_) nanoparticles were simply mixed together to prepare a novel self-enhanced electrochemiluminescence (ECL) composite of Ru-SiO_2_@PEI. The hollow Ru-SiO_2_@PEI nanoparticles were used to build an ECL immunosensor for the analysis of neuron specific enolase (NSE). PEI not only assembled on the surface of Ru-SiO_2_ nanoparticles through the electrostatic interaction to act as co-reactant for Ru(bpy)_3_^2+^ ECL, but also provided alkaline condition to etch the Ru-SiO_2_ nanoparticles to form the hollow Ru-SiO_2_@PEI nanoparticles with porous shell. The unique structure of the Ru-SiO_2_@PEI nanoparticles loaded both a large amount of Ru(bpy)_3_^2+^ and its co-reactant PEI at the same time, which shortened the electron-transfer distance, thereby greatly enhanced the luminous efficiency and amplified the ECL signal. The developed immunosensor showed a wide linear range from 1.0 × 10^−11^ to 1.0 × 10^−5^ mg mL^−1^ with a low detection limit of 1.0 × 10^−11^ mg mL^−1^ for NSE. When the immunosensor was used for the determination of NSE in clinical human serum, the results were comparable with those obtained by using enzyme-linked immunosorbent assay (ELISA) method. The proposed method provides a promising alternative for NSE analysis in clinical samples.

Nowadays, cancer is one of the most threatening diseases for human beings^1^. Thus, the sensitive analysis of tumor biomarkers became greatly important since the level of biomarkers could provide useful information for early diagnosis and disease surveillance. Neuron specific enolase (NSE), a sensitive and reliable tumor marker for small cell lung cancer (SCLC)^2^, could be used to early diagnosis and assess the patient’s recovery progress. Therefore, many methods have been proposed for the detection of NSE, including electrochemical immunoassay^1^,^3^,^4^,^5^, fluorescence immunoassay^6^,^7^, chemiluminescence immunoassay^8^,^9^ etc^10^,^11^,^12^,^13^,^14^. However, developing new sensitive and simple method for the analysis of NSE is still important and meaningful.

As a powerful analytical method, electrochemiluminescence (ECL) has been widely used in environmental monitoring^15^,^16^, food safety^17^, bioanalysis^18^,^19^,^20^,^21^,^22^,^23^,^24^,^25^, and so forth^26^,^27^ due to its high sensitivity, simple set-up and absence of background optical signal. Among the ECL luminophores, Ru(bpy)_3_^2+^ and its derivatives are the most extensively studied luminophores for their high ECL efficiency and good stability in aqueous solution. Ru(bpy)_3_^2+^-doped silica (Ru-SiO_2_) nanoparticles have attracted much attention due to that each Ru-SiO_2_ nanoparticle contained a large amount of Ru(bpy)_3_^2+^ molecules, which could greatly enhance the ECL signal. The Ru-SiO_2_ nanoparticles have been used to construct ECL sensor for DNA analysis^28^,^29^, immunoassay^30^,^31^, cell test^32^,^33^ and so on^34^,^35^,^36^. Since luminophore itself produce low ECL signal, co-reactant such as tripropylamine (TPA) is usually added to the ECL system to enhance the ECL intensity and improve the analytical performance. However, adding co-reactant might lead to a more complex assay system and increase the analytical steps to some extent^37^. Therefore, self-enhanced ECL composite was developed to greatly enhance the ECL signal and simplify the analysis process at the same time. Self-enhanced ECL composite made luminophore and its co-reactant exist in the same composite. In this way, the electron-transfer distance between the luminophore and its co-reactant was shortened and the luminous efficiency could be greatly enhanced^38^. So far, several self-enhanced ECL composites have been proposed and excellent results were obtained^38^,^39^,^40^,^41^. For example, Zhuo *et al.* linked Ru(II) with the co-reactant poly(ethylenimine) (PEI) to prepare a self-enhance ECL composite of Ru(II)-PEI and it was used to build an immunosensor for the analysis of apurinic/apyrimidinic Endonuclease 1^39^. Wang *et al.* immobilized Ru(II) with the co-reactant polyamidoamine dendrimer (PAMAM) on palladium nanowires and used the self-enhance composite to construct carcinoembryonic antigen (CEA) immunosensor^40^. To further expand the application of the simple and sensitive self-enhanced ECL sensor, preparing new and efficient self-enhanced composite is great important.

Herein, we prepared a novel self-enhanced ECL composite by simply combining PEI with Ru-SiO_2_ nanoparticles for the first time, and the obtained hollow Ru-SiO_2_@PEI nanoparticles were used to build an ECL immunosensor for the analysis of NSE. During the interaction, PEI assembled on the surface of the Ru-SiO_2_ nanoparticles through electrostatic interaction to work as co-reactant to greatly enhance the ECL signal. At the same time, PEI provided an alkaline condition to etched the Ru-SiO_2_ to form the hollow Ru-SiO_2_@PEI nanoparticles with porous shell, which was beneficial for the reaction between PEI and Ru(bpy)_3_^2+^. Since a large amount of Ru(bpy)_3_^2+^ and its co-reactant were loaded in the composite at the same time, strong ECL signal was produced. The proposed immunosensor based on the self-enhanced ECL composites showed wide linear range and low detection limit for NSE. The analytical results were consistent with those obtained from the enzyme-linked immunosorbent assay (ELISA) method when the immunosensor was used for the detection of NSE in clinical human serum. The proposed method showed great promise for the NSE analysis in clinical samples.

## Materials and Reagents

Neuron-specific enolase (NSE), NSE antibody (anti-NSE), NSE ELISA kits and Carcinoembryonic antigen (CEA) were purchased from Linc-Bio Science Co. Ltd (Shanghai, China). Triton X-100, bovine serum albumin (BSA), and alpha fetoprotein (AFP) were supplied by Beijing Dingguo Biotechnology Co., Ltd. (Beijng, China). Tetraethyl orthosilicate (TEOS), Tris (2, 2-bipyridyl) dichlororuthenium(II) hexahydrate (Ru(bpy)_3_Cl_2_.6H_2_O), Poly(ethylenimine) (PEI) and HAuCl_4_ were bought from Aldrich. Cyclohexane and ammonium hydroxide (25–28 wt%) were supplied by Beijing Chemical Reagent Factory (Beijing, China). 1-hexanol was offered by Sinopharm Chemical Reagent Co. Ltd (Shanghai, China). Na_2_HPO_4_ and NaH_2_PO_4_ were used to prepare phosphate buffer solutions (PBS). Washing buffer was 0.05% (w/v) Tween-20 in 0.01 M PBS (pH 7.4). All aqueous solutions in the experiments were prepared with doubly distilled water. Human serum samples were obtained from First Affiliated Hospital of Jilin University. Use of serum samples was approved by First Affiliated Hospital of Jilin University. All experiments were performed in accordance with the approved guidelines and regulations. Informed consent was obtained from all subjects.

### Apparatus

MPI-E ECL analyzer (Xi’An Remax Electronic Science & Technology Co. Ltd., China) was used to record the ECL responses. A voltage of 800 V was supplied to the photomultipliertube (PMT). CV behaviors were measured on a CHI832 voltammetric analyzer (Shanghai Chenhua Apparatus Inc., China). Modified GCE electrode, a platinum wire and an Ag/AgCl (saturated KCl) electrode were used as working electrode, counter electrode and reference electrode, respectively. UV-visible spectra were recorded on Cary 50 UV-vis spectrophotometer (Varian, America). AUTOLAB PGSTAT302N was used to record the electrochemical impedance Spectroscopy (EIS). Transmission electron microscopy (TEM) was conducted using a JEOL JEM-1011 electron microscope.

### Preparation of Ru-SiO_2_@PEI nanoparticles

Firstly, Ru-SiO_2_ nanoparticles were prepared according to a previous literature^42^. Briefly, 7.08 mL of TritonX-100, 7.2 mL of 1-hexanol, 30 mL of cyclohexane and 1.36 mL of water were mixed together to form a homogeneous solution under stirring. Then, 320 μL of 0.1 M Ru(bpy)_3_^2+ ^ aqueous solution and 400 μL of TEOS were added. Afterwards, 240 μL of NH_4_OH was added to initiate the polymerization reaction. After reacting for 24 hours, the Ru-SiO_2_ nanoparticles were isolated by adding acetone to the mixture. Followed by washing with ethanol and water for several times to remove any surfactant molecules, the nanoparticles were suspended in 15 mL water for further experiments.

Ru-SiO_2_@PEI nanoparticles were synthesized by adding 10 mL of 20 mg mL^−1^ PEI to 10 mL of 0.5 mg mL^−1^ Ru-SiO_2_ nanoparticles solution and stirred for 12 hours. After centrifuging and washing with water for several times, Ru-SiO_2_@PEI nanoparticles were obtained. The nanoparticles for different PEI concentration or reaction time were prepared in the same way.

### Fabrication of the ECL immunosensor

**Figure 1:**
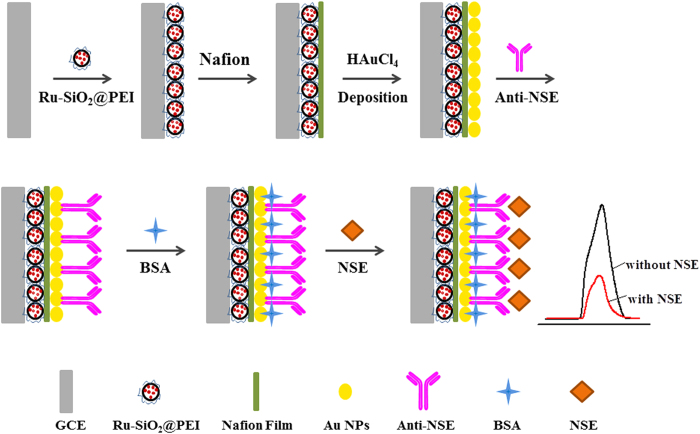
The fabrication process of the proposed immunosensor.

The fabrication process of the immunosensor was exhibited in Fig. 1. Firstly, GCE (3 mm) was polished with 0.3 μm and 0.05 μm alumina powders to obtain mirror-like surface, followed by sonicating in ethanol and water thoroughly and drying at room temperature. 5 μL of Ru-SiO_2_@PEI nanoparticles was dropped onto the pretreated GCE and dried in air. To avoid the ECL composite falling down from the electrode, 2 μL of Nafion was dropped on the electrode to form a Nafion film. Then, the modified electrode was immersed into HAuCl_4_ (1%) solution and electrochemical deposited with constant potential of −0.2 V for 15 s to obtain Au NPs layer. Subsequently, anti-NSE was immobilized on the electrode by soaking the electrode in 80 μL of anti-NSE solution at 4 °C for 24 h. Thereafter, the electrode was immersed in 80 μL of 1% BSA at 37 °C for 1 h to block the nonspecific binding sites. Finally, the electrode was incubated with 80 μL of NSE solution at 37 °C for 80 min to capture the NSE through the interaction between the antibody and antigen. After each modified step, the modified electrode was thoroughly washed with washing buffer to remove the physically absorbed species. ECL response of the proposed immunosensor was investigated in 0.1 M PBS (pH 7.5).

## Results and Discussion

### Characteristics of Ru-SiO_2_ and Ru-SiO_2_@PEI nanoparticles

**Figure 2:**
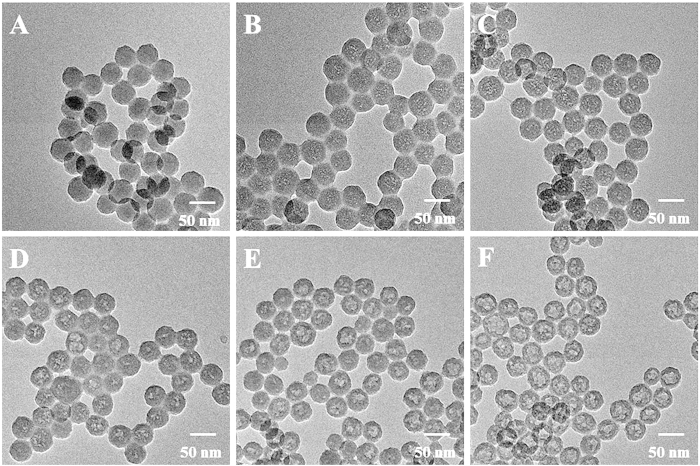
TEM images of (**A**) Ru-SiO_2_ nanoparticles and Ru-SiO_2_@PEI nanoparticles with the reaction time of (**B**) 0.5 h, (**C**) 2 h, (**D**) 6 h, (**E**) 9 h and (**F**) 12 h.

As shown in Fig. 2A, the prepared Ru-SiO_2_ nanoparticles were spherical and uniform in size. When the Ru-SiO_2_ nanoparticles suspension was combined with PEI solution, hollow Ru-SiO_2_@PEI nanoparticles were obtained (Fig. 2F). To understand the formation process of the hollow structure, Ru-SiO_2_@PEI nanoparticles with different reaction time were investigated (Fig. 2B–F). As can be seen, when the reaction time was 0.5 h, many small pores produced inside the nanoparticles. With the increase of reaction time, these small pores merged into a larger cavity and eventually generated the hollow nanoparticles. It’s well-know that SiO_2_ could be etched in alkaline solution by breaking the Si-O-Si bond^43^. During the interaction between Ru-SiO_2_ nanoparticles and PEI, on one hand, PEI could assemble on the surface of Ru-SiO_2_ nanoparticles through electrostatic interaction. As shown in Figure S1, the Ru-SiO_2_ nanoparticles were negatively charged while the Ru-SiO_2_@PEI nanoparticles were positively charged, which suggested that PEI assembled on the surface of Ru-SiO_2_ nanoparticles successfully. On the other hand, the presence of protonated amine groups in the PEI chain provided an alkaline condition, which resulted in the etching of Ru-SiO_2_ nanoparticles^44^. Since the PEI on the surface of the Ru-SiO_2_ nanoparticles could work as protecting agent to prevent the dissolution of the Si-O-Si bond near the outer surface^44^,^45^, as a result, the interior of the Ru-SiO_2_ was etched and eventually formed the hollow Ru-SiO_2_@PEI nanoparticles. In addition, the nitrogen adsorption-desorption isotherm measurement showed that the surface areas increased from 97.8 to 161.9 m^2^ g^−1^ when the Ru-SiO_2_ nanoparticles interacted with PEI to form the hollow Ru-SiO_2_@PEI nanoparticles. From the pore distribution curve measured by the BJH method (Figure S2), we can see that the pore diameter of Ru-SiO_2_ nanoparticles is about 2 nm in average. While for the Ru-SiO_2_@PEI nanoparticles, the ratio of some larger pores with average diameter about 4 nm increased due to the etching of Ru-SiO_2_ nanoparticles. Moreover, when different concentrations of PEI were interacted with Ru-SiO_2_ nanoparticles for 12 h, the hollow-structured nanoparticles were obtained successfully (Figure S3), indicating that the concentration of PEI in the studied range has little effect on the formation of the hollow structure.

**Figure 3:**
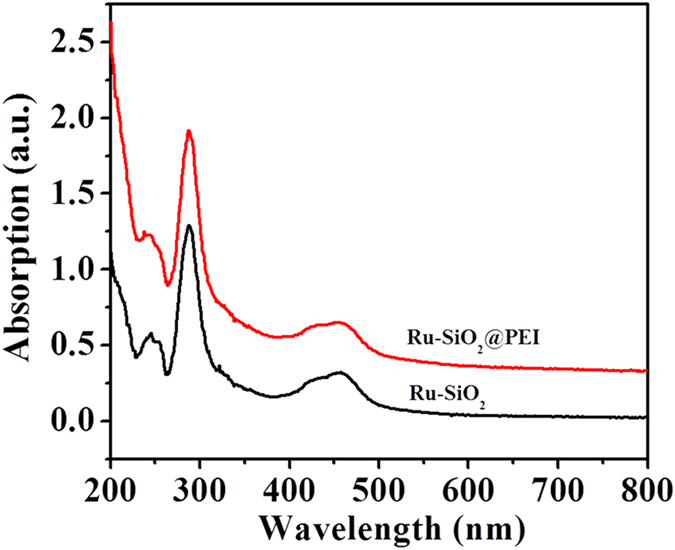
UV-vis absorption spectra of Ru-SiO_2_ and Ru-SiO_2_@PEI nanoparticles.

The UV-vis absorption spectra of Ru-SiO_2_ and Ru-SiO_2_@PEI nanoparticles were shown in Fig. 3. Two obvious peaks appeared at about 287 nm and 457 nm in the spectrum of Ru-SiO_2_ nanoparticles, which were assigned to ligand-centered transitions and metal-to-ligand charge transfer (MLCT) of Ru(bpy)_3_^2+^ molecule, respectively^46^. This indicated that Ru(bpy)_3_^2+^ molecules were successfully encapsulated in the Ru-SiO_2_ nanoparticles. After reacting with PEI to form the hollow Ru-SiO_2_@PEI nanoparticles, there was no obvious change in the UV spectrum, which suggested that the Ru(bpy)_3_^2+^ molecules still maintained in the Ru-SiO_2_@PEI nanoparticles.

### Electrochemistry and ECL behavior

**Figure 4:**
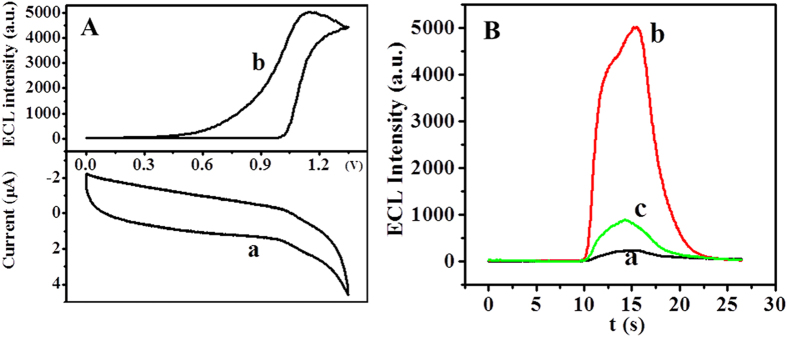
(**A**) Cyclic voltammograms (a) and ECL–potential curve (b) of Ru-SiO_2_@PEI/GCE in 0.1 M PBS (pH 7.5). (**B**) ECL response of (a) Ru-SiO_2_/GCE, (b) Ru-SiO_2_@PEI/GCE in 0.1 M PBS (pH 7.5) and (c) Ru-SiO_2_/GCE in 0.1 M PBS (pH 7.5) containing of 20 mg mL^−1^ PEI. Scan rate: 100 mV s^−1^. Scan potential: 0~1.35 V.

5 μL of 0.5 mg mL^−1^ Ru-SiO_2_@PEI nanoparticles were coated on the GCE electrode (Ru-SiO_2_@PEI/GCE) and the modified electrode was scanned in 0.1 M PBS (pH = 7.5). The cyclic voltammograms (CV) and ECL–potential curve (Fig. 4A) showed that the characteristic redox of Ru(bpy)_3_^2+^ with an oxidation peak appeared near 1.1 V (curve a) and high ECL signal was produced (curve b), which indicated that Ru(bpy)_3_^2+^ molecules in the Ru-SiO_2_@PEI nanoparticles still maintained its properties. The luminescence began around 1.0 V and reached to its maximum value at 1.1 V, which was consistent with the oxidation potential of Ru(bpy)_3_^2+^, suggesting that the oxidation of Ru(bpy)_3_^2+^ played an important role in the process of ECL. In addition, compared with Ru-SiO_2_ nanoparticles modified electrode (Ru-SiO_2_/GCE) (Fig. 4B, curve a), the ECL intensity of Ru-SiO_2_@PEI/GCE increased about 25 times (Fig. 4B, curve b). This great signal enhancement can be attributed to that PEI with amine groups could work as co-reactant to enhance the ECL of Ru(bpy)_3_^2+ ^38. Furthermore, when the Ru-SiO_2_/GCE was scanned in 0.1 M PBS (pH 7.5) containing of 20 mg mL^−1^ PEI (Fig. 4B, curve c), the ECL intensity was enhanced about 5 times, which indicated that PEI could indeed enhance the ECL of Ru(bpy)_3_^2+^. However, the signal enhancement is still much lower than that of Ru-SiO_2_@PEI/GCE. This can be explained that both a large amount of Ru(bpy)_3_^2+^ and its co-reactant PEI were loaded in the Ru-SiO_2_@PEI nanoparticles at the same time so that the electron-transfer distance was shortened, by which the luminous efficiency was enhanced and resulted in the high ECL response^36^. In a word, the prepared Ru-SiO_2_@PEI nanoparticles produced the highest ECL signal by self-enhancement. According to the electrochemistry and ECL behaviors of the Ru-SiO_2_@PEI nanoparticles and the previous reports^38^,^39^, we proposed the possible ECL mechanism of the Ru-SiO_2_@PEI nanoparticles as below:


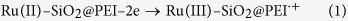



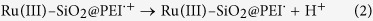



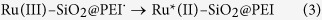



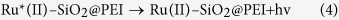


### Characterization of the immunosensor

**Figure 5:**
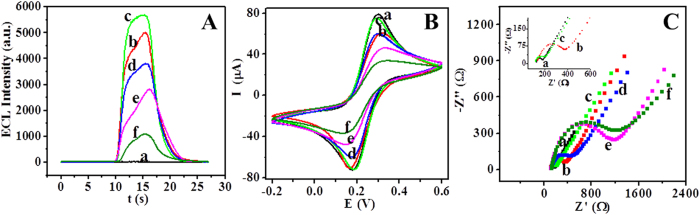
(**A**) ECL, (**B**) CV and (**C**) EIS responses of (a) GCE, (b) Ru-SiO_2_@PEI/GCE (c) Au/Ru-SiO_2_@PEI/GCE, (d) anti-NSE/Au/Ru-SiO_2_@PEI/GCE, (e) BSA/anti-NSE/Au/Ru-SiO_2_@PEI/GCE, (f) NSE/BSA/anti-NSE/Au/Ru-SiO_2_@PEI/GCE. The ECL responses were measured in 0.1 M PBS (pH 7.5); Scan rate: 100 mV s^−1^. Scan potential: 0~1.35 V. CV and EIS were conducted in 0.1 M KCl solution containing of 5.0 mM [Fe(CN)_6_]^3−/4−^.

As indicated above, Ru-SiO_2_@PEI nanoparticles could produce high ECL signal without any extra addition. Thus, we constructed a self-enhanced ECL immunosensor for the analysis of NSE. And then, the fabrication process of the immunosensor was firstly investigated. Figure 5A showed the ECL responses of each step in 0.1 M PBS (curve a). When Ru-SiO_2_@PEI nanoparticles were coated on GCE, great ECL signal was produced (Fig. 5A, curve b). After electrodeposition of Au NPs on the electrode, the ECL intensity was enhanced (Fig. 5A, curve c), since the good conductivity of Au NPs could increase the electron transfer on the electrode and amplified the ECL signal. When anti-NSE was assembled on the electrode via the interaction between the Au NPs and the amino groups of anti-NSE, ECL intensity decreased about 1800 a.u. (Fig. 5A, curve d), which was attributed to that the electronically insert feature of antibody hindered the electron transfer on the electrode and lead to the decrease in ECL response. In addition, ECL signal decreased again after BSA blocked the non-specific site (Fig. 5A, curve e). Finally, when NSE antigen was immobilized on the electrode through the interaction between antigen and antibody, ECL intensity decreased further (Fig. 5A, curve f). These results indicated that each step was constructed successfully, and also suggested that the proposed immunosensor could be used to highly sensitive detection of target antigen based on the decrease of the ECL signal.

CVs responses of each step in 0.1 M KCl containing 5.0 mM [Fe(CN)_6_]^3−/4−^ were displayed in Fig. 5B. The bare GCE exhibited a pair of reversible redox peaks (Fig. 5B, curve a). After Ru-SiO_2_@PEI nanoparticles were coated on the electrode, the peak current decreased due to the poor conductivity of Ru-SiO_2_@PEI nanoparticles (Fig. 5B, curve b). Subsequently, the peak current obviously increased when conductive Au NPs were electrodeposited on the electrode (Fig. 5B, curve c). However, the peak current decreased consecutively when the electrode assembled anti-NSE, BSA and NSE successively (Fig. 5B, curve d–f), owing to that the electron insulted protein molecules blocked the electron transfer on the electrode surface. At the same time, these changes in CV confirmed that anti-NSE, BSA and NSE were immobilized on the electrode successfully.

Electrochemical impedance spectroscopy (EIS) was also conducted to evaluate the fabrication process of the immunosensor (Fig. 5C). When the low conductivity of Ru-SiO_2_@PEI nanoparticles were coated on GCE, electron transfer resistance (R*et*) increased (Fig. 5C, curve b), while the electrodeposition of Au NPs resulted in decreased R*et* (Fig. 5C, curve c). However, R*et* increased consecutively after the successively immobilization of electron insulted anti-NSE, BSA and NSE on the electrode (Fig. 5C, curve d–f). These results were consistent with ECL and CV response.

### Optimization of experimental parameter

To obtain good analytical performance, several experimental parameters, including the concentration of Ru-SiO_2_@PEI, the concentration of the anti-NSE, incubation time between antibody and antigen and pH of the detection solution were optimized. Firstly, the effect of Ru-SiO_2_@PEI concentration on the ECL intensity was shown in Figure S4A. The ECL intensity increased with the increase of Ru-SiO_2_@PEI concentration from 0.1 to 0.5 mg mL^−1^, and it began to decrease thereafter. This was due to that with the increase concentration of Ru-SiO_2_@PEI, more ECL molecules were immobilized on the electrode and produced high ECL signal. However, the ECL emission might be absorbed or scattered by too much ECL molecules^42^. Therefore, 0.5 mg mL^−1^ of Ru-SiO_2_@PEI was used to construct the sensor.

The concentration of the anti-NSE during the construction of the immunosensor was optimized. As shown in Figure S4B, the ECL decreased with the increase of the concentration of the anti-NSE from 0.1 to 1 μg mL^−1^, and it began to stabilize with higher concentration, which indicated the saturated immobilization of anti-NSE on the electrode. Thus, we used 1 μg mL^−1^ anti-NSE to build the immunosensor.

The incubation time between antigen and antibody was an important factor influencing the ECL signal. As indicated in Figure S4C, the ECL intensity decreased with longer incubation time and reached a plateau after 80 min, which showed a saturated binding of NSE on the electrode. Thus, 80 min of incubation time was selected for the sensor.

In addition, pH value effect was also investigated. As exhibited in Figure S4D, the ECL signal increased with pH value from 6.0 to 7.5 and it began to decrease with higher pH value. According to the ECL mechanism of the system, higher pH value was favorable for the deprotonation which resulted in high ECL signal. However, too high pH value might lead to decomposition of some species^42^. Hence, pH 7.5 was used in the experiment.

### Analytical performance of the immunosensor

**Figure 6:**
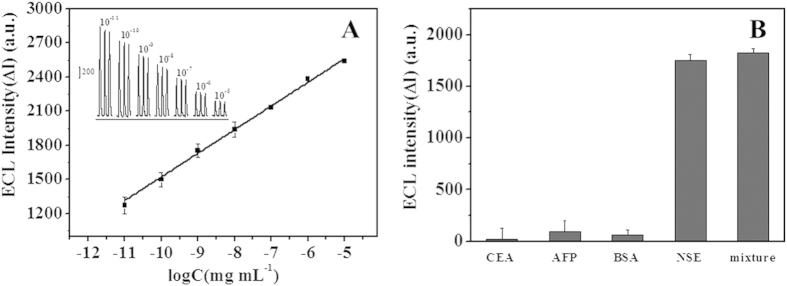
(**A**) Calibration curve of decrease value of ECL intensity (ΔI) to logarithmic NSE concentration. Insert: ECL− time curves of the immunosensor incubating with 1.0 × 10^−11^ mg mL^−1^, 1.0 × 10^−10^ mg mL^−1^, 1.0 × 10^−9^ mg mL^−1^, 1.0 × 10^−8^ mg mL^−1^, 1.0 × 10^−7^ mg mL^−1^, 1.0 × 10^−6^ mg mL^−1^ and 1.0 × 10^−5^ mg mL^−1^ NSE. (**B**) ECL responses of the proposed immunosensor incubating with 2.0 × 10^−8^ mg mL^−1^ of CEA, AFP, BSA, 1.0 × 10^−9^ mg mL^−1^ of NSE and their mixture. The ECL response was determined in 0.1 M PBS (pH 7.5); Scan rate: 100 mV s^−1^. Scan potential: 0~1.35 V.

The analytical performance of the immunosensor was investigated by incubating the immunosensor in NSE solution with different concentrations under the optimal conditions. As shown in Fig. 6A, the ECL intensity decreased with higher concentration of NSE, which due to that the electron insulted NSE hindered the electron transfer on the electrode. The decrease value of ECL intensity (ΔI = I_0_ − I, I_0_ stands for the ECL intensity without NSE and I is the ECL intensity with NSE.) was proportional to the logarithm of NSE concentration in the range from 1.0 × 10^−11^ to 1.0 × 10^−5^ mg mL^−1^ with a detection limit of 1.0 × 10^−11^ mg mL^−1^. The linear regression equation is ΔI = 207.51 × log C_NSE_ + 3599.03 with a correlation coefficient of 0.992. The comparison of analytical performance of the proposed immunosensor with previous reports was listed in Table 1. It can be found that the present work showed lower detection limit and wider liner rang over other approaches.

The excellent analytical performances of the proposed method were attributed to the high ECL intensity produced by the self-enhanced Ru-SiO_2_@PEI nanoparticles. Since both a large amount of Ru(bpy)_3_^2+^ and its co-reactant PEI were loaded on the Ru-SiO_2_@PEI nanoparticles at the same time, the ECL signal was greatly amplified and resulted in the good analytical performances.

AFP, CEA and BSA were used as interference agents to evaluate the specificity of the immunosensor. As shown in Fig. 6B, the ECL responses of pure AFP, CEA and BSA were negligible compared with that of NSE. What’s more, the ECL signal of the mixture of AFP, CEA, BSA and NSE was similar to that of the NSE. These results demonstrated that the proposed immunosensor possessed good specificity for the analysis of NSE. In addition, the relative standard deviation (RSD) for five different immunosensors was 4.4%, which indicated good fabrication reproducibility. Furthermore, the RSD for ten continuous scan of an immunosensor was 5.4% and the ECL signal could still maintain 84% of the initial intensity after one month, which suggested good stability of the immunosensor.

### Analysis of NSE in clinical human serum

To investigate the practical application of the proposed immunosensor, it was used to the detection of NSE in clinical human serum samples. The assay results were compared with the reference value obtained by the commercial ELISA kits. As shown in Table 2, the relative deviation between the two methods was range from −2.16 to 8.11%, which indicated that the developed immunosensor had potential application in the analysis of NSE in clinical samples.

## Conclusion

A novel self-enhanced electrochemiluminescence immunosensor for the analysis of NSE was developed based on hollow Ru-SiO_2_@PEI nanoparticles. The unique structure of Ru-SiO_2_@PEI nanoparticles as well as high loading of Ru(bpy)_3_^2+^ and PEI greatly enhanced the luminous efficiency and resulted in high ECL intensity. The developed immunosensor exhibited seven orders of magnitude linear range and 1.0 × 10^−11^ mg mL^−1^ detection limit for the analysis of NSE. The immunosensor was successfully used to the analysis of clinical human serum samples, and the results are consistent with that obtained by the commercial ELISA kits. The proposed method provides a promising alternative for NSE antigen analysis in clinical samples.

## Additional Information

**How to cite this article**: Zhou, L. *et al.* A novel self-enhanced electrochemiluminescence immunosensor based on hollow Ru-SiO_2_@PEI nanoparticles for NSE analysis. *Sci. Rep.* **6**, 22234; doi: 10.1038/srep22234 (2016).

## Supplementary Material

Supplementary Information

## Figures and Tables

**Table 1:** Comparison of analytical performance of proposed immunosensor with other methods.

Method	Linear range (mg mL^−1^)	Detection limit (mg mL^−1^)	Reference
Label-less immunoassay based upon an alternating current impedance protocol	1.0 × 10^−9^ −5.0 × 10^−8^	5.0 × 10^−10^	4
Multiplexing determination by immunocapture LC-MS/MS		2.1 × 10^−7^	10
Electrocatalytic oxidation of DA based on NiHCFNPs to amplify electrochemical signal	1.0 × 10^−9^ −1.0 × 10^−4^	3.0 × 10^−10^	1
Simultaneous detection of two lung cancer biomarkers using dual-color fluorescence quantum dots	3.0 × 10^−6^ −1.0 × 10^−4^	1.0 × 10^−6^	6
Photoelectrochemical immunosensor based on ZnCdHgSe quantum dots/polymerized ionic liquid hybrid film	1.0 × 10^−9^ −1.0 × 10^−4^	2.0 × 10^−10^	11
Microfluidic competitive enzyme immunoassay based on chemiluminescence resonance energy transfer	4.2 × 10^−7^ −4.4 × 10^−5^	2.1 × 10^−7^	8
Fluorescent and electrochemical detection based on CaCO_3_/AuNCs hybrid spheres	5.0 × 10^−9^ −1.0 × 10^−6^ 5.0 × 10^−10^ −2.0 × 10^−6^	2.0 × 10^−9^ 1.0 × 10^−10^	7
ECL immunosensor based on Ru-SiO_2_@PEI nanoparticles	1.0 × 10^−11^ −1.0 × 10^−5^	1.0 × 10^−11^	This work

**Table 2:** Assay results of NSE level in clinical serum samples using the proposed ECL immunosensor and ELISA method.

Sample	ECL immunosensor (ng mL^−1^, n = 6)	ELISA (ng mL^−1^)	Relative Standard Deviation (%)
1	39.27	39.95	−1.72
2	50.05	47.27	5.87
3	100.55	93.01	8.11
4	110.43	112.87	−2.16

## References

[b1] Han J., Zhuo Y., Chai Y. Q., Yuan Y. L. & Yuan R. Novel electrochemical catalysis as signal amplified strategy for label-free detection of neuron-specific enolase. Biosens. Bioelectron. 31, 399–405 (2012). 22169815 10.1016/j.bios.2011.10.055

[b2] Oremek G. M., Sauer-Eppel H. & Bruzdziak T. H. Value of tumour and inflammatory markers in lung cancer. Anticancer Res. 27, 1911–1915 (2007). 17649794

[b3] Lu W. *et al.* An electrochemical immunosensor for simultaneous multiplexed detection of two lung cancer biomarkers using Au nanoparticles coated resin microspheres composed of l-tryptophan and caffeic acid. Ionics 21, 1141–1152 (2015).

[b4] Barton A. C., Davis F. & Higson S. P. J. Labeless immunosensor assay for the stroke marker protein neuron specific enolase based upon an alternating current impedance protocol. Anal. Chem. 80, 9411–9416 (2008). 19007247 10.1021/ac801394d

[b5] Wang L., Liu N. & Ma Z. Novel gold-decorated polyaniline derivatives as redox-active species for simultaneous detection of three biomarkers of lung cancer. J. Mater. Chem. B 3, 2867–2872 (2015). 10.1039/c5tb00001g 32262415

[b6] Li H., Cao Z., Zhang Y., Lau C. & Lu J. Simultaneous detection of two lung cancer biomarkers using dual-color fluorescence quantum dots. Analyst 136, 1399–1405 (2011). 21279241 10.1039/c0an00704h

[b7] Peng J. *et al.* Calcium carbonate-gold nanocluster hybrid spheres: synthesis and versatile application in immunoassays. Chem. -Eur. J. 18, 5261–5268 (2012). 22422592 10.1002/chem.201102876

[b8] Yang T., Vdovenko M., Jin X., Sakharov I. Y. & Zhao S. Highly sensitive microfluidic competitive enzyme immunoassay based on chemiluminescence resonance energy transfer for the detection of neuron-specific enolase. Electrophoresis 35, 2022–2028 (2014). 24723253 10.1002/elps.201300630

[b9] Fu X. *et al.* Chemiluminescence enzyme immunoassay using magnetic nanoparticles for detection of neuron specific enolase in human serum. Anal. Chim. Acta. 722, 114–118 (2012). 22444542 10.1016/j.aca.2012.02.007

[b10] Torsetnes S. B. *et al.* Multiplexing determination of small cell lung cancer biomarkers and their isovariants in serum by immunocapture LC-MS/MS. Anal. Chem. 86, 6983–6992 (2014). 24945626 10.1021/ac500986t

[b11] Yu X. *et al.* White-light-exciting, layer-by-layer-assembled ZnCdHgSe quantum dots/polymerized ionic liquid hybrid film for highly sensitive photoelectrochemical immunosensing of neuron specific enolase. Anal. Chem. 87, 4237–4244 (2015). 25790014 10.1021/ac504456w

[b12] Cheng S., Hideshima S., Kuroiwa S., Nakanishi T. & Osaka T. Label-free detection of tumor markers using field effect transistor (FET)-based biosensors for lung cancer diagnosis. Sens. Actuators, B 212, 329–334 (2015).

[b13] Fu X., Xu K., Ye J., Chen J. & Feng X. Glucoamylase-labeled nanogold flowers for *in situ* enhanced sensitivity of a glucometer-based enzyme immunoassay. Anal. Methods 7, 507–512 (2015).

[b14] Raluca-Ioana S. S., Ionela R. C., Carmen C. S. & Marius B. Nanostructured materials detect epidermal growth factor receptor, neuron specific enolase and carcinoembryonic antigen. Nanoscale 7, 15689–15694 (2015). 26350155 10.1039/c5nr04476f

[b15] Jiang D. *et al.* Anchoring AgBr nanoparticles on nitrogen-doped graphene for enhancement of electrochemiluminescence and radical stability. Chem. Commun. 51, 4451–4454 (2015). 10.1039/c4cc09926e 25679205

[b16] Du X. *et al.* An ON1-OFF-ON2 electrochemiluminescence response: combining the intermolecular specific binding with a radical scavenger. Chem. Commun. 51, 11236–11239 (2015). 10.1039/c5cc04029a 26076759

[b17] Hu T., Li T., Yuan L., Liu S. & Wang Z. Anodic electrogenerated chemiluminescence of quantum dots: size and stabilizer matter. Nanoscale 4, 5447–5453 (2012). 22837021 10.1039/c2nr31324c

[b18] Hu T., Liu X., Liu S., Wang Z. & Tang Z. Toward understanding of transfer mechanism between electrochemiluminescent dyes and luminescent quantum dots. Anal. Chem. 86, 3939–3946 (2014). 24690085 10.1021/ac5004823

[b19] Li H. *et al.* A nanobody-based electrochemiluminescent immunosensor for sensitive detection of human procalcitonin. Analyst 139, 3718–3721 (2014). 24931592 10.1039/c4an00626g

[b20] Gao F., Lei J. & Ju H. Label-free surface-enhanced raman spectroscopy for sensitive DNA detection by DNA-mediated silver nanoparticle growth. Anal. Chem. 85, 11788–11793 (2013). 24171654 10.1021/ac4032109

[b21] Lou J., Liu S., Tu W. & Dai Z. Graphene quantums dots combined with endonuclease cleavage and bidentate chelation for highly sensitive electrochemiluminescent DNA biosensing. Anal. Chem. 87, 1145–1151 (2015). 25523862 10.1021/ac5037318

[b22] Zhou J. *et al.* Electrochemiluminescence imaging for parallel single-cell analysis of active membrane cholesterol. Anal. Chem. 87, 8138–8143 (2015). 26213787 10.1021/acs.analchem.5b00542

[b23] Xia H., Li L., Yin Z., Hou X. & Zhu J. Biobar-coded gold nanoparticles and DNAzyme-based dual signal amplification strategy for ultrasensitive detection of protein by electrochemiluminescence. ACS Appl. Mater. Interfaces 7, 696–703 (2015). 25475153 10.1021/am506980d

[b24] Huang T., Meng Q. & Jie G. Silver nanowires-based signal amplification for CdSe quantum dots electrochemiluminescence immunoassay. Biosens. Bioelectron. 66, 84–88 (2015). 25460886 10.1016/j.bios.2014.11.011

[b25] Wu L., Ma C., Zheng X., Liu H. & Yu J. Paper-based electrochemiluminescence origami device for protein detection using assembled cascade DNA-carbon dots nanotags based on rolling circle amplification. Biosens. Bioelectron. 68, 413–20 (2015). 25618373 10.1016/j.bios.2015.01.034

[b26] Xu Y., Yin X. B., He X. W. & Zhang Y. K. Electrochemistry and electrochemiluminescence from a redox-active metal-organic framework. Biosens. Bioelectron. 68, 197–203 (2015). 25569877 10.1016/j.bios.2014.12.031

[b27] Yu Y., Lu C. & Zhang M. Gold nanoclusters@Ru(bpy)(3)(2+)-layered double hydroxide ultrathin film as a cathodic electrochemiluminescence resonance energy transfer probe. Anal. Chem. 87, 8026–8032 (2015). 26159772 10.1021/acs.analchem.5b02208

[b28] Wu M. S., He L. J., Xu J. J. & Chen H. Y. RuSi@Ru(bpy)(3)(2+)/Au@Ag2S nanoparticles electrochemiluminescence resonance energy transfer system for sensitive DNA detection. Anal. Chem. 86, 4559–4565 (2014). 24707967 10.1021/ac500591n

[b29] Bae S. W. *et al.* A doubly signal-amplified DNA detection method based on pre-complexed [Ru(bpy)(3)](2+)-doped silica nanoparticles. Chem. -Eur. J. 16, 11572–11575 (2010). 20803581 10.1002/chem.201001255

[b30] Shao K. *et al.* Stretch–stowage–growth strategy to fabricate tunable triply-amplified electrochemiluminescence immunosensor for ultrasensitive detection of pseudorabies virus antibody. Anal. Chem. 86, 5749–5757 (2014). 24845014 10.1021/ac500175y

[b31] Li J., Guo L. R., Gao W., Xia X. H. & Zheng L. M. Enhanced electrochemiluminescence efficiency of Ru(II) derivative covalently linked carbon nanotubes hybrid. Chem. Commun. 7545–7547; doi: 10.1039/B916007H (2009). 20024274

[b32] Wu Y. *et al.* Signal amplification cytosensor for evaluation of drug-induced cancer cell apoptosis. Anal. Chem. 84, 1894–1899 (2012). 22242898 10.1021/ac202672x

[b33] Chen Z., Liu Y., Wang Y., Zhao X. & Li J. Dynamic evaluation of cell surface N-Glycan expression via an electrogenerated chemiluminescence biosensor based on concanavalin a-integrating gold-nanoparticle-modified Ru(bpy)(3)(2+)-doped silica nanoprobe. Anal. Chem. 85, 4431–4438 (2013). 23560766 10.1021/ac303572g

[b34] Chen L. *et al.* A sensitive aptasensor for adenosine based on the quenching of Ru(bpy)(3)(2+)-doped silica nanoparticle ECL by ferrocene. Chem. Commun. 46, 7751–7753 (2010). 10.1039/c0cc03225e 20852786

[b35] Jia T. T. *et al.* Electrogenerated chemiluminescence ethanol biosensor based on alcohol dehydrogenase functionalized Ru(bpy)(3)(2+) doped silica nanoparticles. Biosens. Bioelectron. 25, 263–267 (2009). 19616932 10.1016/j.bios.2009.06.030

[b36] Lv X. *et al.* Electrochemiluminescence modified electrodes based on RuSi@Ru(bpy)32+loaded with gold functioned nanoporous CO/Co3O4 for detection of mycotoxin deoxynivalenol. Biosens. Bioelectron. 70, 28–33 (2015). 25791464 10.1016/j.bios.2015.03.020

[b37] Liao N. *et al.* Reagent less electrochemiluminescent detection of protein biomarker using graphene-based magnetic nanoprobes and poly-L-lysine as co-reactant. Biosens. Bioelectron. 45, 189–194 (2013). 23500362 10.1016/j.bios.2013.02.005

[b38] Wang H., He Y., Chai Y. & Yuan R. A super intramolecular self-enhanced electrochemiluminescence immunosensor based on polymer chains grafted on palladium nanocages. Nanoscale 6, 10316–10322 (2014). 25072965 10.1039/c4nr02808b

[b39] Zhuo Y. *et al.* Ultrasensitive apurinic/apyrimidinic endonuclease 1 immunosensing based on self-enhanced electrochemiluminescence of a Ru(II) complex. Anal. Chem. 86, 1053–1060 (2014). 24328308 10.1021/ac403019e

[b40] Wang H., Yuan Y., Chai Y. & Yuan R. Self-enhanced electrochemiluminescence immunosensor based on nanowires obtained by a green approach. Biosens. Bioelectron. 68, 72–77 (2015). 25562733 10.1016/j.bios.2014.12.016

[b41] Zhao M. *et al.* Triple quenching of a novel self-Enhanced Ru(II) complex by hemin/G-quadruplex DNA zymes and its potential application to quantitative protein detection. Anal. Chem. 87, 7602–7609 (2015). 26135920 10.1021/acs.analchem.5b01671

[b42] Zhang L. & Dong S. Electrogenerated chemiluminescence sensors using Ru(bpy)(3)(2+) doped in silica nanoparticles. Anal. Chem. 78, 5119–5123 (2006). 16841937 10.1021/ac060451n

[b43] Park S. J., Kim Y. J. & Park S. J. Size-dependent shape evolution of silica nanoparticles into hollow structures. Langmuir 24, 12134–12137 (2008). 18834158 10.1021/la8028885

[b44] Zhang L. *et al.* General route to multifunctional uniform yolk/mesoporous silica shell nanocapsules: a platform for simultaneous cancer-targeted imaging and magnetically guided drug delivery. Chem. -Eur. J. 18, 12512–12521 (2012). 22907903 10.1002/chem.201200030

[b45] Zhang Q., Zhang T., Ge J. & Yin Y. Permeable silica shell through surface-protected etching. Nano Lett. 8, 2867–2871 (2008). 18698725 10.1021/nl8016187

[b46] Innocenzi P., Kozuka H. & Yoko T. Fluorescence properties of the Ru(bpy)(3)(2+) complex incorporated in sol-gel-derived silica soating films. J. Phys. Chem. B 101, 2285–2291 (1997).

