# Supplementary material for: A novel self-enhanced electrochemiluminescence immunosensor based on hollow Ru-SiO2@PEI nanoparticles for NSE analysis
Source: Sci Rep. 2016 Feb 26;6:22234. doi: 10.1038/srep22234 (PMC4768085; doi:10.1038/srep22234)
Supplement: Supplementary Information [file srep22234-s1.pdf]

Supporting Information

A novel self-enhanced electrochemiluminescence immunosensor based on hollow Ru-SiO_2@PEI nanoparticles for NSE analysis

Limin Zhou[1,2]， Jianshe Huang [1], Bin Yu[1,3], Tianyan You[1*]

[1]State Key Laboratory of Electroanalytical Chemistry, Changchun Institute of Applied Chemistry, Chinese Academy of Sciences, Changchun, 130022, China

[2]University of the Chinese Academy of Sciences, Beijing, 100049, China

[3]National Engineering Laboratory for AIDS Vaccine, School of Life Sciences, Jilin University, Changchun, 130012, China

Corresponding Author

* Tel: +86-431-85262850.

Fax: +86-431-85262850.

E-mail address: youty@ciac.ac.cn.

1. Zeta potential of Ru-SiO_2 and Ru-SiO_2@PEI nanoparticles

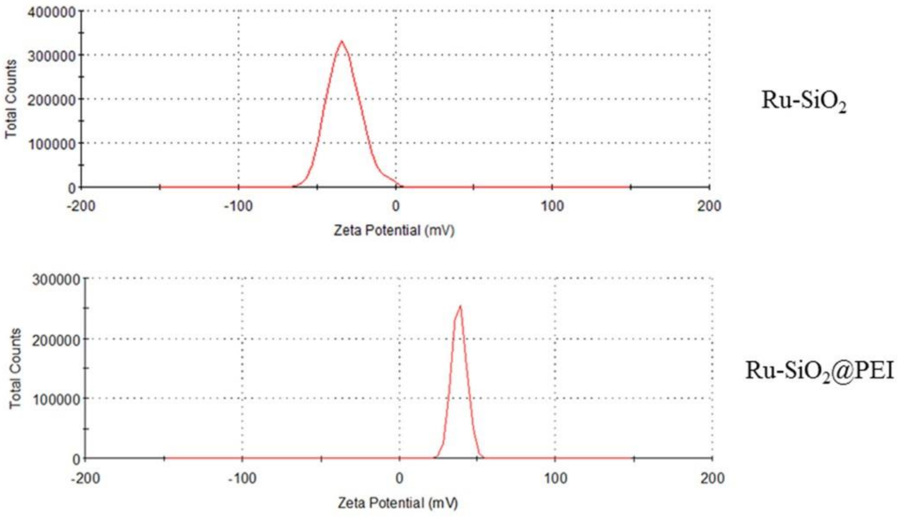

Figure S1 Zeta potential of Ru-SiO_2 and Ru-SiO_2@PEI nanoparticles

2. Nitrogen adsorption-desorption isotherm and BJH pore size distribution of Ru-SiO_2 and Ru-SiO_2@PEI nanoparticles

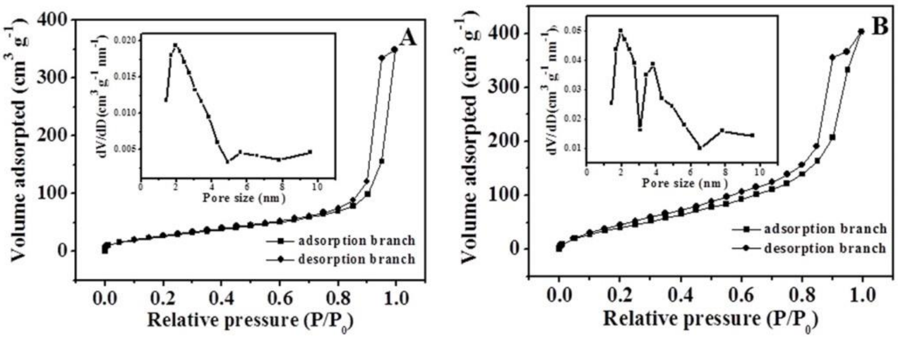

Figure S2 Nitrogen adsorption-desorption isotherm for Ru-SiO_2 and Ru-SiO_2@PEI nanoparticles. Inset: BJH pore diameter distribution.

3. TEM images of Ru-SiO_2@PEI nanoparticles by interacting Ru-SiO_2 nanoparticles with different concentration of PEI

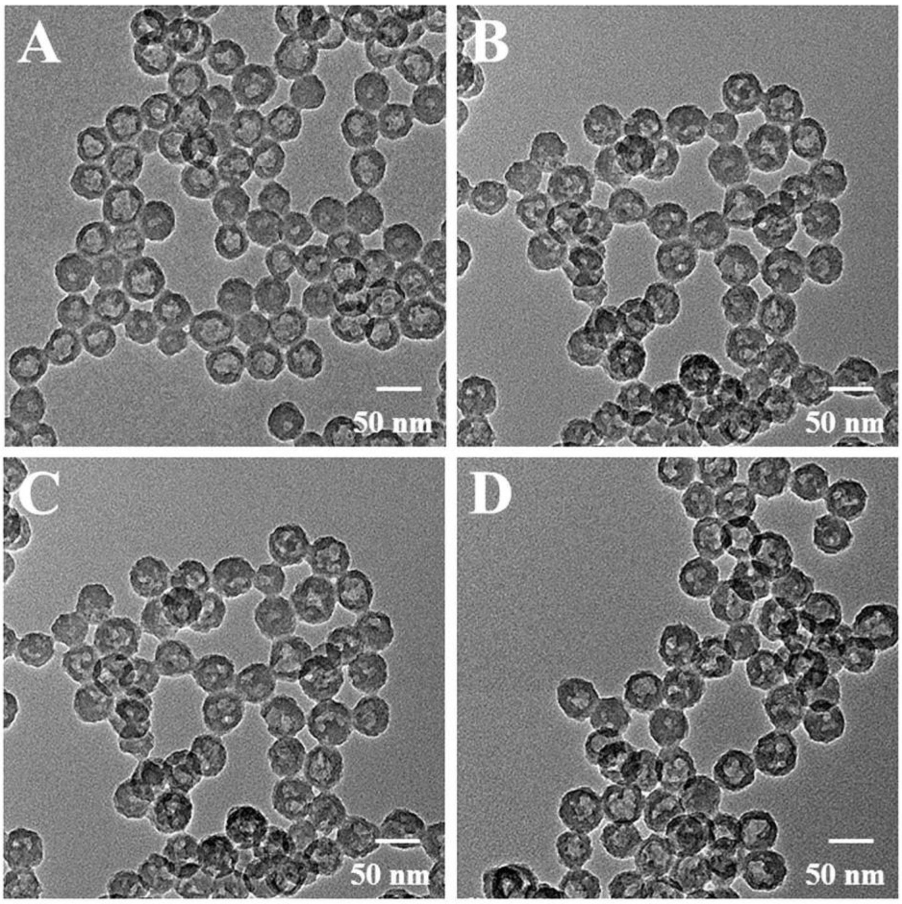

Figure S3 TEM images of Ru-SiO_2@PEI nanoparticles by interacting Ru-SiO_2 nanoparticles with (A) 5 mg mL^{-1}, (B) 15 mg mL^{-1}, (C) 25 mg $mL^{-1}$and (D) 40 mg mL^{-1} PEI for 12h.

4. Optimization of experimental parameter

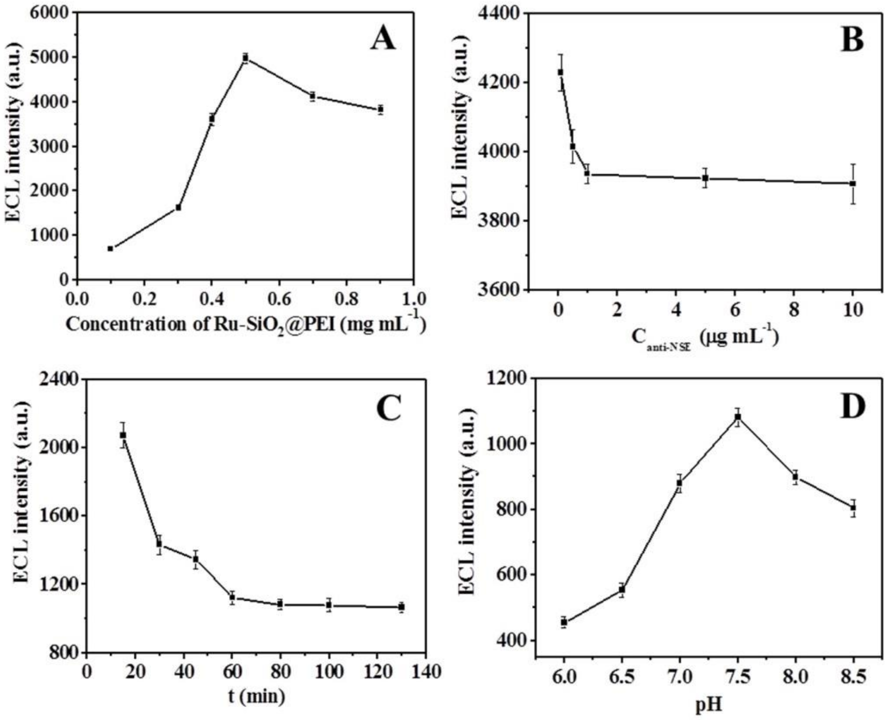

Figure S4 The effects of (A) concentration of Ru-SiO_2@PEI nanoparticles, (B) the concentration of the anti-NSE, (C) incubation time and (D) pH value on the ECL intensity. The immunosensor was incubated with 1.0×10^{-9} mg mL^{-1} NSE antigen. The ECL response was determined in 0.1M PBS (pH 7.5); Scan rate: 100 mV s^{-1}. Scan potential: 0~1.35V
